# Sustained rubber hand illusion after the end of visuotactile stimulation with a similar time course for the reduction of subjective ownership and proprioceptive drift

**DOI:** 10.1007/s00221-021-06211-8

**Published:** 2021-09-15

**Authors:** Z. Abdulkarim, Z. Hayatou, H. H. Ehrsson

**Affiliations:** 1 Department of Neuroscience, Karolinska Institutet, Stockholm, Sweden; 2 Université Paris-Saclay, CNRS, Institut Des Neurosciences Paris-Saclay, 91190 Gif-sur-Yvette, France

**Keywords:** Multisensory integration, Embodiment, Proprioception, Rubber hand illusion

## Abstract

The rubber hand illusion is a perceptual illusion in which participants experience an inanimate rubber hand as their own when they observe this model hand being stroked in synchrony with strokes applied to the person’s real hand, which is hidden. Earlier studies have focused on the factors that determine the elicitation of this illusion, the relative contribution of vision, touch and other sensory modalities involved and the best ways to quantify this perceptual phenomenon. Questionnaires serve to assess the subjective feeling of ownership, whereas proprioceptive drift is a measure of the recalibration of hand position sense towards the rubber hand when the illusion is induced. Proprioceptive drift has been widely used and thought of as an objective measure of the illusion, although the relationship between this measure and the subjective illusion is not fully understood. Here, we examined how long the illusion is maintained after the synchronous visuotactile stimulation stops with the specific aim of clarifying the temporal relationship in the reduction of both subjective ownership and proprioceptive drift. Our results show that both the feeling of ownership and proprioceptive drift are sustained for tens of seconds after visuotactile stroking has ceased. Furthermore, our results indicate that the reduction of proprioceptive drift and the feeling of ownership follow similar time courses in their reduction, suggesting that the two phenomena are temporally correlated. Collectively, these findings help us better understand the relationships of multisensory stimulation, subjective ownership, and proprioceptive drift in the rubber hand illusion.

**Supplementary Information:**

The online version contains supplementary material available at 10.1007/s00221-021-06211-8.

## Introduction

The rubber hand illusion is a perceptual illusion in which participants experience an inanimate rubber hand as their own (Botvinick and Cohen 1998). The illusion is brought about by placing a rubber hand in front of the participant and synchronously stroking the participant’s hand and the rubber hand at corresponding anatomical locations, while the participant’s real hand is hidden from sight. After a period of such synchronous visuotactile stimulation, the participants start to experience that the rubber hand is their own hand and start to refer the touch to the viewed rubber hand (Botvinick and Cohen 1998). Illusory experience has been measured through subjective questionnaires. However, more objective measures have been developed. The skin conductance response (SCR) is measured by threatening the rubber hand with a sharp object after the induction of the illusion while measuring the associated changes in conductance in the skin, which is interpreted as the autonomic stress response to the threat (Armel and Ramachandran 2003; Petkova and Ehrsson 2009). Proprioceptive drift is measured by having the participants indicate the perceived location of the real hidden hand before and after the illusion and then calculating the difference between those measurements (Botvinick and Cohen 1998; Tsakiris and Haggard 2005). If the illusion is induced, the participants will display a larger recalibration of hand position sense towards the rubber hand in the illusion condition compared to the control condition (Abdulkarim and Ehrsson 2016; Botvinick and Cohen 1998; Guterstam et al. 2011).

Since its introduction, the proprioceptive drift measure has been widely used as a measure of the rubber hand illusion, probably because it is easy to administer, does not require any advanced technical equipment, and has been shown to correlate with illusory experience (Botvinick and Cohen 1998; Costantini and Haggard 2007; H. Henrik Ehrsson et al. 2005; Guterstam et al. 2013; Kalckert and Ehrsson 2014; Longo et al. 2008; Samad et al. 2015; Tsakiris et al. 2006). However, recent findings have suggested that proprioceptive drift and the illusory experience of the rubber hand illusion do not always correlate (Holle et al. 2011; Riemer et al. 2015; Rohde et al. 2011). In particular, studies have demonstrated that participants can display a proprioceptive drift without experiencing any changes in body ownership (Holmes et al. 2004, 2006; Makin et al. 2008) and that changes in body ownership have been reported without being accompanied by significant changes in proprioceptive drift (Abdulkarim and Ehrsson 2016; Rohde et al. 2013). These studies have thus indicated that proprioceptive drift and the sense of ownership are two independent processes in the rubber hand illusion, raising the question of the exact relationship between the two. In our previous study, we showed that experimentally manipulating the physical location of the participants’ real hand during the induction of the rubber hand illusion without the participant noticing caused changes in the proprioceptive drift but did not change the subjective ratings of the illusion (Abdulkarim and Ehrsson 2016). This observation speaks against proprioceptive drift being a causal factor in generating the rubber hand illusion, but the subjective illusion could still cause proprioceptive drift, and the two could be correlated.

In the current study, we use a different approach to examine the relationship between proprioceptive drift and the subjective rubber hand illusion. Unlike previous studies that have focused on the elicitation of the rubber hand illusion by repeated stroking and the subsequent period of relatively stable illusion experience as the dynamic visuotactile stimulations continue to be delivered, we investigated what happens when the visuotactile stimulation stops once the illusion has been evoked. How long does it take before the illusory perception “switches back” to veridical perception? Do proprioceptive drift and subjective ownership follow similar time courses during this period of illusion loss? Anecdotally, anyone who has tested the rubber hand illusion knows that the illusion does not immediately vanish when the synchronous stroking stops. It seems to be maintained for at least a few seconds afterwards; indeed, many rubber hand illusion studies use this period to probe the illusion (Botvinick and Cohen 1998; Ehrsson et al. 2007; Reader and Ehrsson 2019; Tsakiris and Haggard 2005). However, how long the subjective illusion and proprioceptive drift persist beyond this initial period of a few seconds is unknown and the basic question of how the rubber hand illusion decays when the synchronous visuotactile stimulation stops is a theoretically interesting one in its own right. Visuotactile stroking has been considered critical for the induction of the illusion (Guterstam et al. 2019; Makin et al. 2008; Tsakiris 2010), so according to these views, one might expect the illusion to disappear when the synchronized stroking stops. However, a critical difference from the illusion elicitation phase is that once the participants experience that the rubber hand is part of their own body, the synchronous visuotactile stimulation might no longer be as important. During the elicitation phase, the correlated visuotactile stimulations provide sensory evidence in favor of the rubber hand being one’s own, but the absence of such visuotactile correlations does not provide evidence against the illusion when the illusion has already been elicited. Therefore, a gradual loss of the rubber hand illusion in the poststimulation period must be driven either by the disparity between proprioceptive and visual signals regarding the location of one’s hand or by prior beliefs regarding the constituents of one’s body. Thus, without synchronized stroking providing a continuous source of sensory evidence in favor of the illusion, these factors would gradually build up until the body representation would update back to veridical bodily perception. We thus hypothesized that the rubber hand illusion would be maintained for some time after the visuotactile stimulation stopped and then gradually decayed, although we did not have any specific predictions regarding the precise time course.

Our second aim was to use this period of hypothesized gradual illusion loss to investigate the relationship between proprioceptive drift and subjective illusion in terms of their temporal dynamics. In particular, we examined possible differences in how long the subjective sense of rubber hand ownership and proprioceptive drift is maintained, how fast the two illusion measures decay, and the specific temporal relationship of these two decay functions. To this end, we compared time series of proprioceptive drift and ownership questionnaire ratings in the period of 0 to 300 s following the end of the visuotactile stimulation. This approach has several advantages compared to earlier studies that typically just correlate the two phenomena after a single measurement at the end of a period of visuotactile stimulation: it allows the probing of more fine-grained changes that only develop over time, as different temporal decay functions would be indicative of different underlying processes, and it allows the relationship of proprioceptive drift and ownership to be examined without the potentially interfering synchronous or asynchronous visuotactile stimulation (Rohde et al 2011). Taking into account the literature described above, which has indicated that proprioceptive drift and subjective illusion ratings are two different phenomena, our working hypothesis was that the temporal dynamics of their decay curves would differ.

## Materials and methods

### Participants and ethics

Participants were recruited using an online advertising platform for recruiting study participants. All but one participant was right handed (assessed with the 10-question version of the Edinburgh handedness inventory (Oldfield 1971)) and had normal or corrected-to-normal vision. The participants were healthy and did not take any medication. A total of 40 naïve participants were recruited for the two experiments (Experiment 1: 20 participants, 11 females, 9 males. Mean age 29.6 years, range 18–55; Experiment 2: 20 participants, 10 females, 10 males. Mean age 31.8 years, range 18–46). The participants were given one cinema ticket for their participation in the study. The number of participants recruited for each experiment was based on previous experiments with the same methods and predicted effect sizes (Abdulkarim and Ehrsson 2016; Guterstam et al. 2011; Holle et al. 2011; Rohde et al. 2011). All experiments were conducted according to the Declaration of Helsinki and approved by the Swedish Ethical Review Authority.

### Setup

**Fig. 1:**
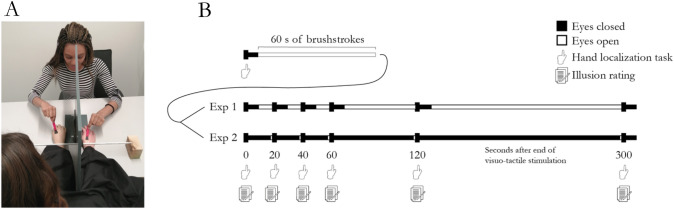
**A** Photograph of the experimental setup. The experimenter, facing the photographer, is seen holding the two small paintbrushes used for the visuo-tactile stimulation. The participant’s real right hand is placed furthest to the right in the picture, hidden from the participant’s view behind the divider. The rubber hand is placed to the left of the divider in full view of the participant. The experimenter induced the rubber hand illusion by synchronously stroking the rubber hand and the participant’s real right hand with two small paintbrushes on corresponding parts and in the same direction. The metal ruler rod was placed over the participant’s wrist in this photograph illustrating the illusion induction phase, but it could easily be moved by the experimenter to a more distal position over the tip of the participant’s index finger before the participants were asked to indicate the position of their right index finger in the hand localization task used to register the proprioceptive drift. **B** Schematic illustration of the procedures for experiments 1 and 2. The hand localization task and illusion ratings were performed in two separate sessions in each experiment. In the proprioceptive drift trials, a baseline hand localization task was performed before the start of the 60 s of visuo-tactile stimulation. Immediately following the visuotactile stimulation, in the proprioceptive drift trials, the participants closed their eyes and performed the hand localization task again (0 s) and then again at 20 s, 40 s, 60 s, 120 s and 300 s after the end of the visuotactile stimulation. In the second part (subjective ratings) of the experiment, no hand localization task was performed; instead, the participant verbally rated the subjective feeling of the rubber hand feeling like their own hand on a seven-point Likert scale at each of the six timepoints. In experiment 1, the participants opened their eyes after they had performed the hand localization task to keep their eyes open as much as possible during the trials, whereas in experiment 2, they kept their eyes as closed throughout the session and only opened them for 1 s immediately prior to performing the hand localization task or the subjective rating at the six specified timepoints

A right-handed male cosmetic prosthetic hand (Ottobock Group, Stockholm, Sweden) was placed in front of the participants. A plastic divider was placed lateral to the rubber hand, behind which the participant’s real hand was placed in a relaxed position. The rubber hand, in view, and the real hand, hidden behind the divider more laterally, were placed 15 cm apart (Fig. 1, panel A). A piece of black cloth covered the participant’s right shoulder and extended towards the rubber hand, thus giving the impression that the rubber hand is continuous with the body. The plastic divider had a magnetically closed hatch in it, which allowed it to be opened and closed. Through this hatch, a metal rod with ruler marks was placed. The metal rod extended from medially to the rubber hand to laterally to the participant’s real hidden hand, passing over the tip of the index finger of the rubber hand as well as the tip of the participant’s real right index finger. This placement of the rod allowed the participant to slide their index finger across both hands when indicating the position of their right index finger to allow the participants to make use of the whole ruler, including locations lateral to the divider and the real hand. Two small paintbrushes (approximately 1 cm wide) were used for visuotactile stimulation (Fig. 1, panel A).

## Design and procedure

### Experiment 1

The experiment was divided into two parts: in the first part, the proprioceptive drift of the rubber hand illusion was registered, and in the second part, the subjective rating of the rubber hand illusion was measured. We chose to divide the experiment into two parts and always register the proprioceptive drift before the subjective illusion ratings, because in the literature, proprioceptive drift has often been administered before the subjective illusion ratings to avoid having statements in the questionnaire bias the participants’ responses in the proprioceptive drift measure, since the questionnaire usually includes statements regarding the position of the hand. The control condition in both experiments was an asynchronous condition in which visuo-tactile stroking was delivered asynchronously on the rubber hand and on the real hand with a delay of approximately 0.5 s.

After their arrival at the testing room, the participant was seated and provided with written information about the experiment as well as instructions about the task they had to perform. A consent form was also given to the participant to sign. The participants were asked to place their left hand on the table and their right hand behind the plastic divider (both symmetrically at the same distance from the body midline). The rubber hand was placed on the other side of the divider in the participant’s field of view. The black cloth was then placed over the participant’s right arm and the space between the rubber hand and the participant’s body as described above.

In the first part, before the induction of the illusion, the participant performed the baseline hand localization task for the proprioceptive drift measure. This preillusion hand localization was performed by asking the participant to close their eyes and slide their left index finger along the horizontal ruler on the table and point to where they felt their right index finger was located. The experimenter asked the participant to close their eyes and placed the participant’s left index finger on the left side of the metal ruler (the starting point on the metal ruler was varied randomly by the experimenter). The participant was instructed to do one swift motion towards their right index finger and then stop and utter the word “here” when they felt that they were holding their left index immediately above their right index finger. The experimenter then noted the value and returned the participant’s left hand to a relaxed position on the table and subsequently asked the participant to open their eyes again. The whole hand localization task procedure, including moving the participant’s left hand to the metal ruler hand back to its relaxed position on the table and the experimenter noting the measurement, took approximately 10–20 s. We used this intermanual pointing task to probe proprioceptive drift, because it has been widely used and reproduced before (Botvinick and Cohen 1998; Abdulkarim and Ehrsson 2016; Guterstam et al. 2013; Kalckert and Ehrsson 2012; Kammers et al. 2006).

The rubber hand illusion was then induced for 60 s by applying synchronous brushstrokes to the participant’s right hand and the rubber hand (Fig. 1, panel B). The brushstrokes were applied to all fingers as well as the dorsum of the hand, and the brushstrokes always started proximally and ended distal to the starting point. Each brushstroke was approximately 3 cm long. The brushstrokes were applied with a regular frequency of approximately 1 Hz. The experimenter took great care to try to apply a similar pattern of visuo-tactile stimulation across all trials by applying brushstrokes to the same fingers and the back of the hand. The only difference between the synchronous and asynchronous conditions was the relative timing of the seen and felt brushstrokes. In the asynchronous condition, the strokes applied to the rubber hand and real hand occurred in an alternating pattern with approximately 0.5 s between the seen and felt strokes.

Immediately after the end of the synchronous visuotactile stimulation, the hand localization task was repeated as described above and then further repeated at 20 s, 40 s, 60 s, 120 s and 300 s after the end of the visuotactile stimulation in every trial. The proprioceptive drift was calculated for each trial by subtracting the prestimulation hand localization from the poststimulation hand localization measurement (for each of the different timepoints) in line with earlier studies (Abdulkarim and Ehrsson 2016; Holle et al. 2011; Rohde et al. 2011). A positive value denotes a proprioceptive drift towards the rubber hand, which is the expected direction of the effect. Proprioceptive drift data from the hand localization task were collected three times per condition in separate trials, and the order of conditions was pseudorandomized to prevent order effects.

In the second part of the experiment, the participant was seated in front of the table with the rubber hand illusion set up as in the first part. The rubber hand illusion was induced for 60 s by applying synchronous brush strokes to the participant’s right hand and the rubber hand. Immediately after the end of the synchronous visuotactile stimulation, the subjective illusion measurement was made and then repeated at 20 s, 40 s, 60 s, 120 s and 300 s after the end of the visuotactile stimulation in every trial. Each measurement was repeated once per condition, and the order of conditions was pseudorandomized (Fig. 1, panel B). The subjective illusion measurement was obtained by asking the participants to rate a statement commonly used to assess the feeling of ownership using a seven-point Likert scale from − 3 to 3, where − 3 corresponds to “completely disagree”, 3 corresponds to “completely agree”, and 0 corresponds to “neither agree nor disagree”. The statement used was “I felt as if the rubber hand were my hand” (Abdulkarim and Ehrsson 2016; Botvinick and Cohen 1998; Kalckert and Ehrsson 2017). The experimenter presented the question verbally, and the participant verbally reported the rating score, which the experimenter quickly noted in a spreadsheet on a laptop.

### Experiment 2

All procedures in experiment 2 were identical to those described for experiment 1 with a few exceptions, as described below. The aim of experiment two was to better control for the amount of time the participant had their eyes open and looked at the rubber hand during the different phases of the testing. We know that visual feedback from looking at the rubber hand can influence illusion strength due to visuoproprioceptive integration (Durgin et al. 2007; Kalckert and Ehrsson 2012; Samad et al. 2015; Trojan et al. 2018; Walsh et al. 2011), even though such effects can be small and the illusion cannot typically be elicited by looking at the rubber hand alone (Guterstam et al. 2019). However, if only viewing the rubber hand can lead to a slower reduction for one of our outcome measures, we theorized this could affect the relative time course of proprioceptive drift and ownership, and therefore, we designed the second experiment to better control for this factor throughout all timepoints sampled from 0 to 300 s.

We also wanted to replicate the key findings from experiment 1. In experiment 1, the participants had their eyes open in between the measurements of the proprioceptive drift and throughout the trials in which the subjective ratings were obtained. However, because the classic proprioceptive drift task we used took some time to administer (each trial lasted between approximately 10 and 20 s), most participants had their eyes closed throughout the measurements at 0, 20, 40 and 60 s, after which there was a pause long enough for them to have time to open their eyes. In experiment 2, by contrast, the participants had their eyes closed starting at the end of the visuo-tactile stimulation and throughout the 300-s-long testing phase. They only opened their eyes for one second (under the careful surveillance and instructions of the experimenter) to view the rubber hand immediately before the subjective ratings were obtained or the hand localization task was performed to make it clear to the participants that they should rate the feeling of ownership over the rubber hand (Fig. 1, panel B).

### Statistical analysis

The first step of the analysis was to analyze at which timepoint the proprioceptive drift or subjective feeling of ownership could be considered no longer significantly present. The proprioceptive drift and subjective illusion ratings in the synchronous condition were compared to the drifts and ratings in the asynchronous condition for each timepoint using Student’s *t* tests. The proprioceptive drift and subjective feeling of ownership were considered absent at the timepoint in which there was no significant difference between the measurements in the synchronous and asynchronous condition.

The second step of the analysis was to analyze the decay of the subjective illusion ratings and proprioceptive drift and compare their time courses. To this end, we first *z*-score normalized the synchronous and asynchronous measures separately for each participant across timepoints for both the subjective illusion ratings and the proprioceptive drift. We then randomized a subpopulation (7 participants), for which we pooled the data for each outcome measure and condition separately. We fitted various curves to this pooled data set (i.e., linear, exponential and polynomial) by first calculating a residual norm cost function and then finding the minima of this function using the function “fminsearch” (Nelder-Mead method) in MATLAB (version 2018b). The starting points entered into the function “fminsearch” were randomized real numbers between 0 and 10, and the function went through ten thousand iterations in the process of finding the minima of the cost function. Once we identified the best fitting curve type, we fitted this curve type to each participant’s individual data and extracted the slopes and intersect as well as the goodness of fit coefficients (*R*^2^). This fitting procedure was repeated 100 times for each participant with randomized starting points to find the parameters yielding the best fit while still conforming to the curve type and form of the best fitting curve. We then compared the coefficients for the “intersect”, decay and constant as well as the goodness of fit between the two outcome measures and conditions using 2 × 2 ANOVAs (proprioceptive drift/subjective illusion ratings & synchronous/asynchronous). We also used the fitted curves to calculate the “half-life” of the outcome measures, namely, the time in seconds it takes for the z-scores at timepoint 0 to decrease by 50 percent and compared these between outcome measures and conditions in a 2 × 2 ANOVA. Furthermore, we compared the intersects, decays, constants and half-lives of the two outcome measures in the synchronous conditions with Bayesian hypothesis testing to examine whether there was any support for the null hypothesis. We also ran a correlation analysis for the intersects, decays, constants and half-lives between the two outcome measures to see if the individual coefficient for one outcome measure could predict the coefficient for the other outcome measure.

In an additional analysis, the same curve selection and fitting procedure was performed for the *z* score normalized synchronous–asynchronous difference for the two outcome measures. The resulting coefficients for the fitted curves were then compared between the two outcome measures using a Student’s *t* test as well as a Bayesian statistical test.

For independent comparisons between the two experiments, we ran 2 × 2x6 ANOVAs for the proprioceptive drift and subjective illusion ratings separately with the factors experiment, synchrony and timing, followed by Mann–Whitney *U* tests for each individual timepoint for the synchronous–asynchronous difference for the proprioceptive drift and subjective illusion ratings separately. The Mann–Whitney *U* test was used due to violations of the assumption of normality in the data. All statistical comparisons were performed using JASP software (version 0.14.1, University of Amsterdam, The Netherlands).

## Results

### Experiment 1

#### Proprioceptive drift

**Fig. 2:**
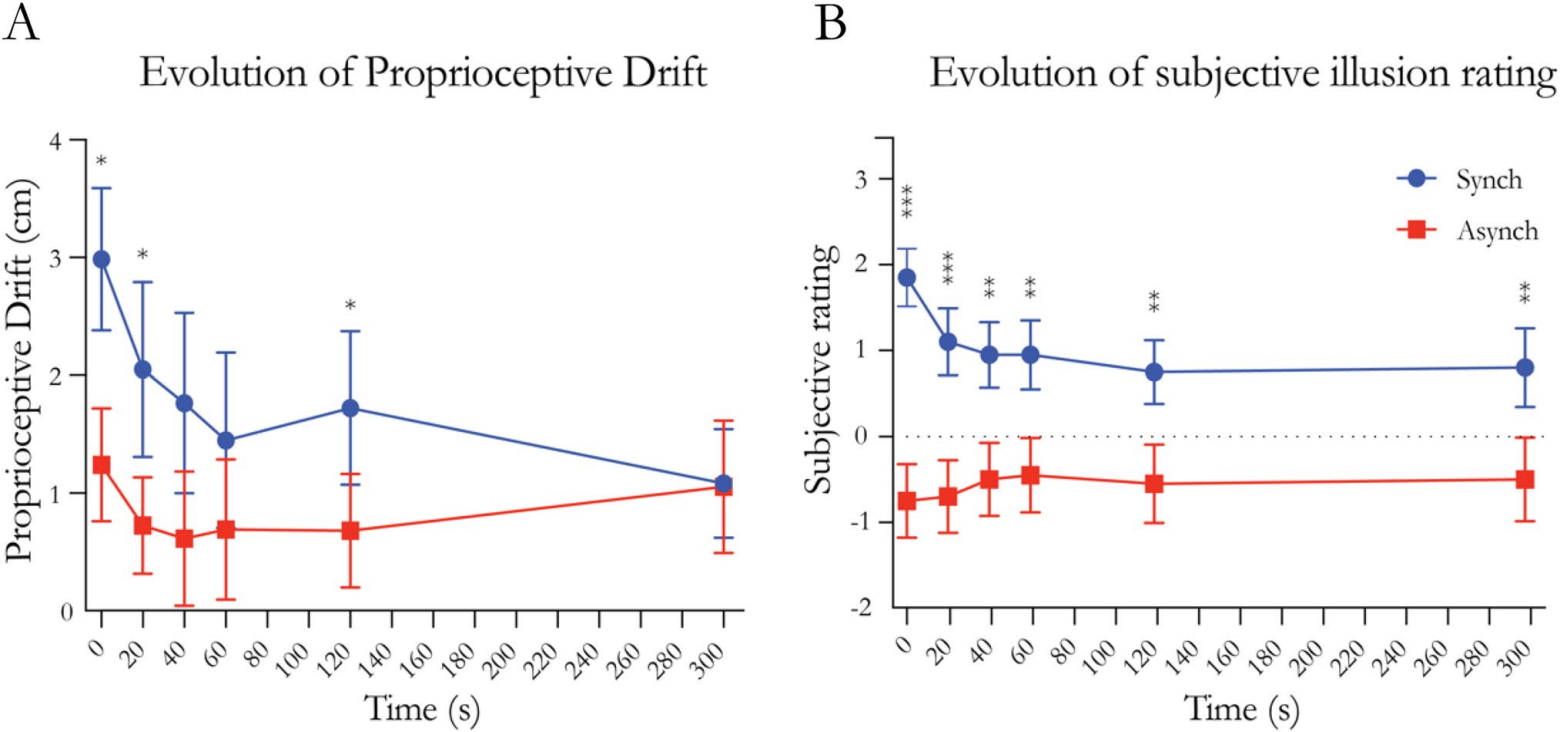
Results from experiment 1. **A** Evolution of the proprioceptive drift following the end of the visuo-tactile stimulation. **B** Evolution of the subjective ratings of the statement “it felt as if the rubber hand were my hand” after the end of the visuotactile stimulation. Timepoint 0 indicates immediately after the end of the visuo-tactile stimulation. Blue lines indicate the synchronous condition, while red lines indicate the asynchronous condition. Dots and squares indicate means, and error bars indicate the SEMs. Stars indicate significance in the comparison between Synch and Async; *indicates *p* < 0.05, **indicates *p* < 0.01, and ***indicates *p* < 0.001

To compare the proprioceptive drift between the synchronous and asynchronous conditions, *t* tests were performed between the synchronous and asynchronous measurements for each timepoint (Fig. 2, panel A). The analysis revealed a significant difference at timepoints 0 s (*t* = 2.442, df = 19, *p* = 0.025, Cohen’s *d* = 0.546), 20 s (*t* = 2.157, df = 19, *p* = 0.044, Cohen’s *d* = 0.482) and 120 s (*t* = 2.334, df = 19, *p* = 0.031, Cohen’s *d* = 0.522). Comparisons at all other timepoints were nonsignificant (Supplementary Table 1).

#### Subjective illusion rating

*T* tests were performed between the synchronous and asynchronous measurements for each timepoint (Fig. 2, panel B). The analysis revealed a significant difference at all timepoints (Supplementary Table 1).

#### Comparing the slopes of the proprioceptive drift and subjective illusion rating

**Fig. 3:**
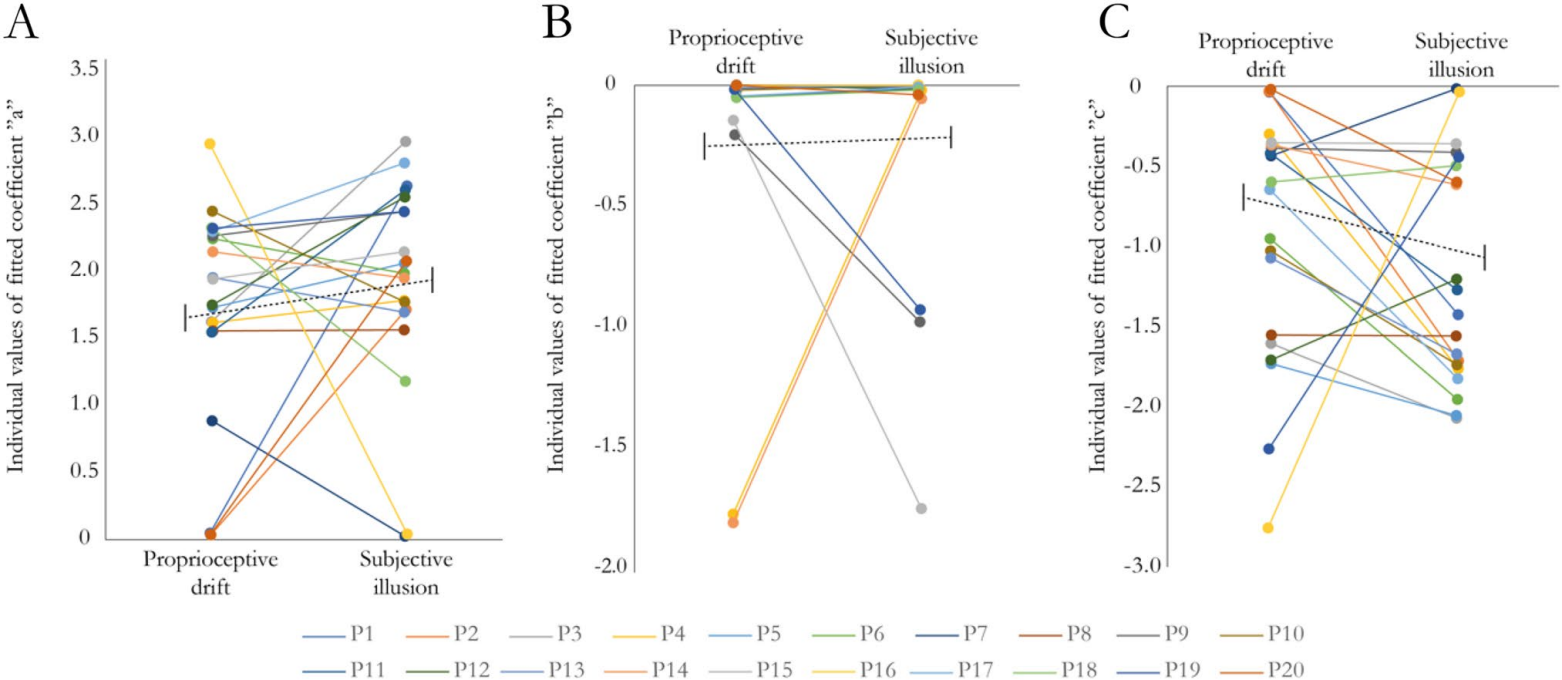
Comparison of the values of the coefficients for the fitted curves (a, b and c in the equation $y\, = \,a\, \times \,e^{bx} \, + \,c$) for the synchronous conditions in experiment 1. Panel **A** illustrates the coefficient “*a*” (intersect), panel **B** the coefficient “*b*” (decay) and panel **C** the coefficient “*c*” (constant). Individual data points and pairwise comparisons are shown for all participants (P1–P20). The black dotted lines illustrate mean and standard error of the mean (SEM). See Supplementary Fig. 1 for individual fitted curves and individual datapoints

**Fig. 4:**
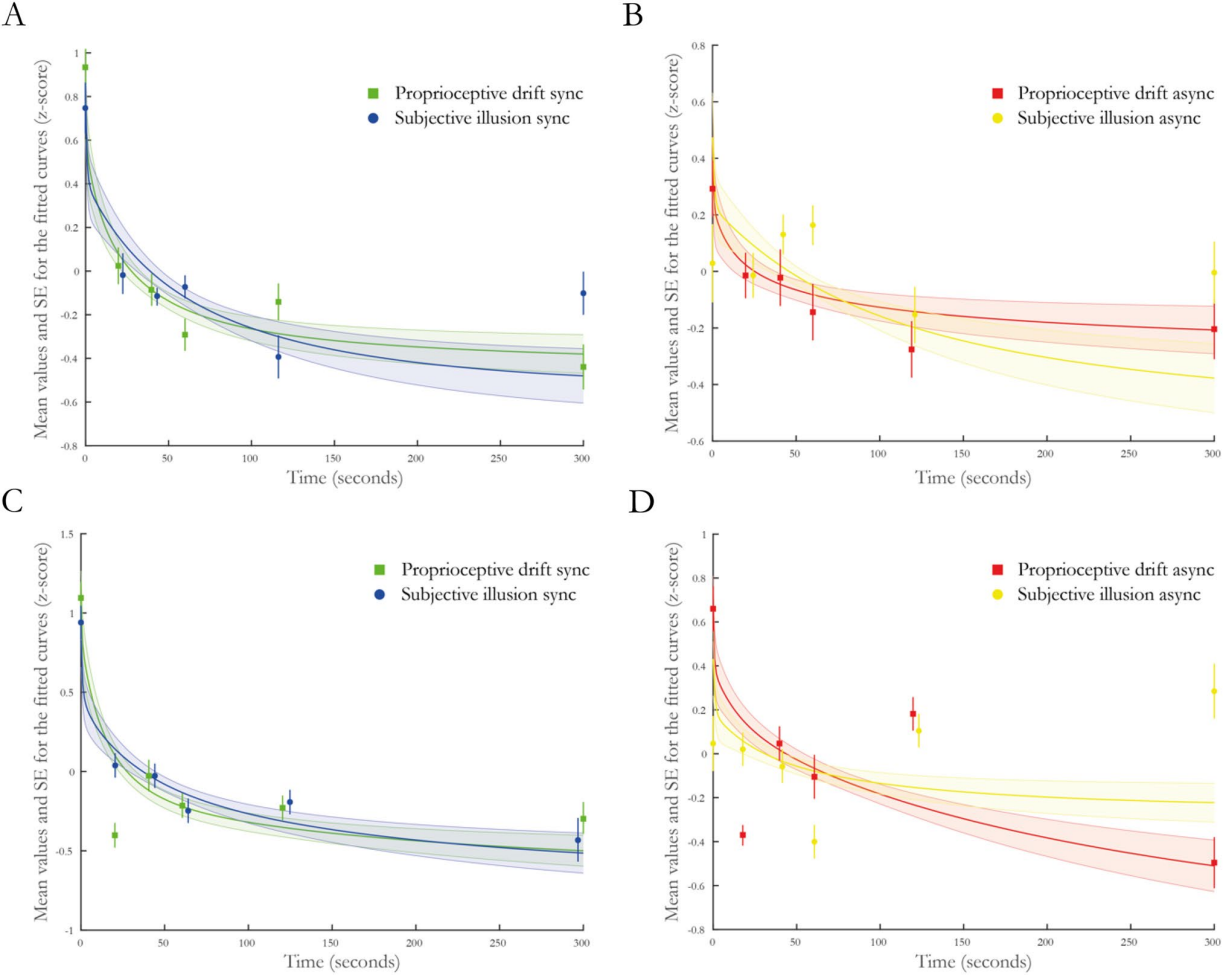
Panels **A** and **B** depict the mean fitted curves from experiment 1, whereas panels **C** and **D** depict the mean fitted curves from experiment 2. The exponential curves represent the mean fitted curve (averaged fitted values) and the shaded areas around them indicate the standard errors. The squares and dots indicate the mean z-scores for each of the six measured timepoints (0, 20, 40, 60, 120 and 300 s), with the error bars indicating the standard error of the mean, *n* = 20 for each experiment. See Supplementary Fig. 1 and Supplementary Fig. 4 for individual datapoints and illustration of the fitted curves

We fitted exponential curves to the Z-scored synchronous and asynchronous measures in the proprioceptive drift and subjective illusion rating, respectively, for each participant individually and extracted the parameter estimates. The fitted exponential curve was in the form of *y* = *a* × *e*^*bx*^ + *c*. Each parameter (a, b and c) was analyzed with a 2 × 2 ANOVA, as were the *R*^2^ values and the half-lives (se methods). To illustrate these results, we have included a figure with the pairwise comparisons of the parameters (a, b and c) of the fitted curves for the synchronous conditions (Fig. 3) as well as a figure displaying the average of the fitted curves overlayed with the *z* scores for all conditions (Fig. 4, panels A and B).

The analysis of the curve fits (*R*^2^ values, see Supplementary Table 2) showed no significant main effect of outcome measure (*F* = 0.833, df = 17, *p* = 0.374, *η*^2^ = 0.021) or synchrony (*F* = 0.813, df = 17, *p* = 0.380, *η*^2^ = 0.014) and no significant interaction (*F* = 1.492, df = 17, *p* = 0.239, *η*^2^ = 0.019). The analysis of the intersect values (‘a’ in the equation $y\, = \,a\, \times \,e^{bx} \, + \,c$) showed no significant main effect of outcome measure (*F* = 0.002, df = 17, *p* = 0.961, *η*^2^ < 0.0001) or synchrony (*F* = 2.861, df = 17, *p* = 0.107, *η*^2^ = 0.041) and no significant interaction (*F* = 1.412, df = 17, *p* = 0.249, *η*^2^ = 0.025). The same pattern of results was observed for the decay coefficient (‘b’ in the equation $y\, = \,a\, \times \,e^{bx} \, + \,c$), with no main effect of outcome measure (*F* = 0.183, df = 17, *p* = 0.673, *η*^2^ = 0.006), synchrony (*F* = 0.457, df = 17, *p* = 0.507, *η*^2^ = 0.05) and no interaction (*F* = 0.288, df = 17, *p* = 0.598, *η*^2^ = 0.003), indicating that the decay of the fitted curves in both outcome measures had similar steepness and temporal dynamics. Finally, the constant values for the fitted exponential decay curves (‘c’ in the equation $y\, = \,a\, \times \,e^{bx} \, + \,c$) similarly showed no significant main effect of outcome measure (*F* = 0.002, df = 17, *p* = 0.964, *η*^2^ < 0.0001), no significant main effect of synchrony (*F* = 0.000194, df = 17, *p* = 0.989, *η*^2^ < 0.0001) and no interaction (*F* = 1.626, df = 17, *p* = 0.218, *η*^2^ = 0.038). In an additional analysis, we calculated the half times of the fitted curves, which takes into account both the intersect, decay coefficient and constant of the exponential decay curve. The analysis revealed no significant main effects (outcome measure: *F* = 1.271, df = 17, *p* = 0.274, *η*^2^ = 0.021, synchrony: *F* = 1.264, df = 17, *p* = 0.275, *η*^2^ = 0.021) or interaction (*F* = 1.387, df = 17, *p* = 0.253, *η*^2^ = 0.023), further strengthening the interpretation that the curves display similar decays between outcome measures. Bayesian hypothesis testing between the proprioceptive drift and subjective illusion ratings for each coefficient and half-life in the synchronous conditions showed weak to moderate support for the null hypothesis with Bayes factors (BF_10_) in the range 0.233–0.382 (Supplementary Table 3). The correlation analyses between the intersect, slope, constants and half-life of the curves for the proprioceptive drift and subjective illusion ratings showed no significant correlation (Supplementary Table 4).

In a supplementary analysis to compare the outcome measures, the synchronous–asynchronous differences were calculated and then z-score normalized. Curves were fitted to the data points as described in the methods section, and the coefficients of the resulting exponential curves on the form of *y* = *a* × *e*^*bx*^ + *c* were compared statistically. The analysis revealed no significant differences, with weak to moderate support for the null hypothesis with Bayes factors (BF_10_) in the range 0.250–0.361 (Supplementary Fig. 2, Panel A, Supplementary Fig. 3, Panel A & Supplementary Table 5).

### Summary

The first step of the analysis indicates that the subjective feeling of ownership persists for up to 300 s after the end of the visuo-tactile stimulation, whereas the proprioceptive drift is no longer significantly different between the synchronous and asynchronous after 40 s. Thus, the illusion is not immediately eliminated when the visuotactile stimulation stops but rather shows substantial persistence. In the second step of the analysis, we show that the proprioceptive drift and the subjective rating display a similar time course with decreasing values over time with similar slopes and half times of their fitted exponential decay curves. Thus, we found no support for our initial hypothesis that proprioceptive drift and subjective ownership should display significantly different time courses of reduction; in contrast, both measures seemed to follow a rather similar general decay function, albeit with the synchronous–asynchronous difference persisting for longer in the subjective illusion ratings.

### Experiment 2

#### Proprioceptive drift

**Fig. 5:**
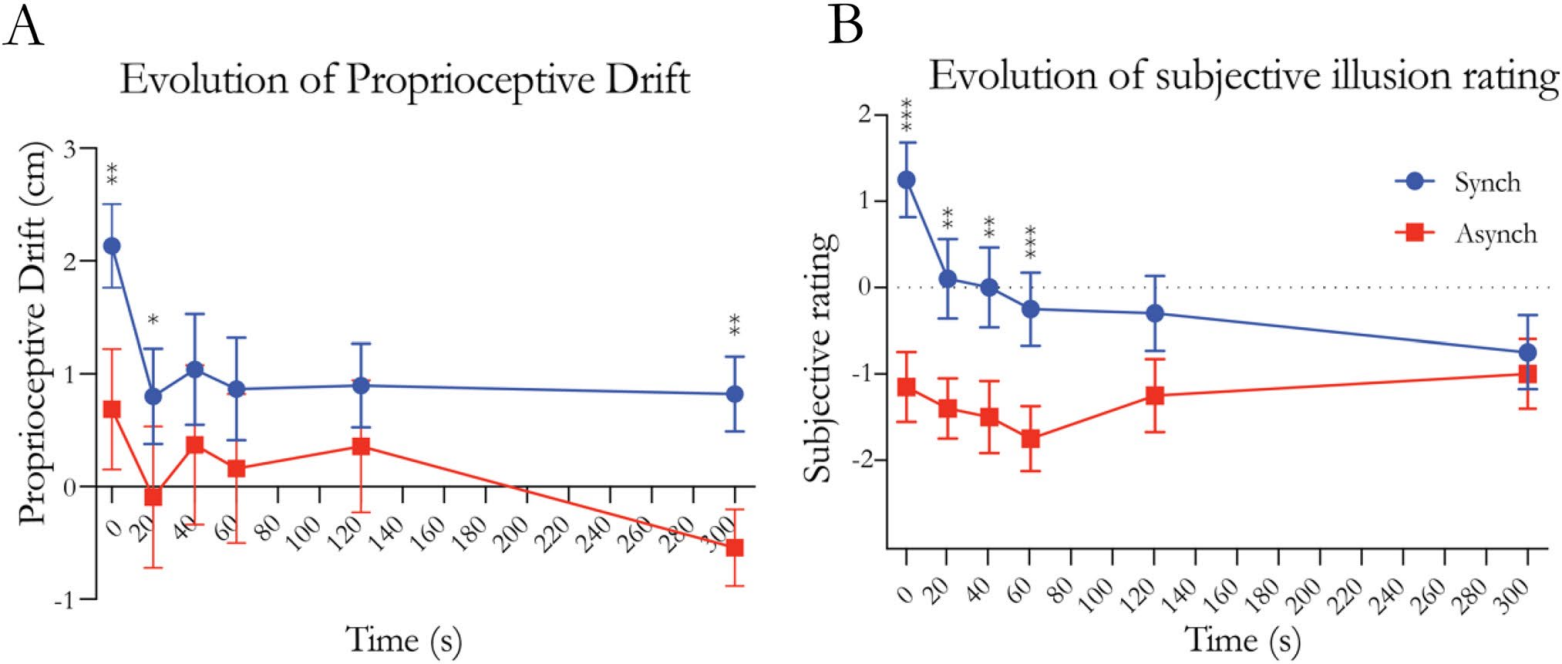
Results from experiment 2. **A** Evolution of the proprioceptive drift following the end of the visuo-tactile stimulation. **B** Evolution of the subjective ratings of the statement “it felt as if the rubber hand were my hand” after the end of the visuotactile stimulation. Timepoint 0 indicates immediately after the end of the visuo-tactile stimulation. Blue lines indicate the synchronous condition, while red lines indicate the asynchronous condition. Dots and squares indicate means, and error bars indicate the SEMs. Stars indicate significance in the comparison between Synch and Async; *indicates *p* < 0.05, **indicates *p* < 0.01, and ***indicates *p* < 0.001

As in experiment 1, a set of *t* tests was performed between the synchronous and asynchronous measurements for each timepoint (Fig. 5, panel A). The analysis revealed a significant difference at timepoints 0 s (*t* = 3.463, df = 19, *p* = 0.003, Cohens’ *d* = 0.774), 20 s (*t* = 2.179, df = 19, *p* = 0.042, Cohen’s *d* = 0.487) and 300 s (*t* = 3.239, df = 19, *p* = 0.004, Cohen’s *d* = 0.724). Comparisons at all other timepoints were nonsignificant (Supplementary Table 6).

#### Subjective rating

*T* tests between the synchronous and asynchronous measurements for each timepoint (Fig. 5, panel B) revealed a significant difference at timepoints 0 s (*t* = 6.439, df = 19, *p* < 0.001, Cohen’s *d* = 1.440), 20 s (*t* = 3.807, df = 19, *p* = 0.001, Cohen’s *d* = 0.851), 40 s (*t* = 3.249, df = 19, *p* = 0.004, Cohen’s *d* = 0.726), and 60 s (*t* = 4.094, df = 19, *p* < 0.001, Cohen’s *d* = 0.916). Comparisons at the 120 s and 300 s timepoints were nonsignificant (Supplementary Table 6).

#### Comparison between proprioceptive drift and subjective ratings

**Fig. 6:**
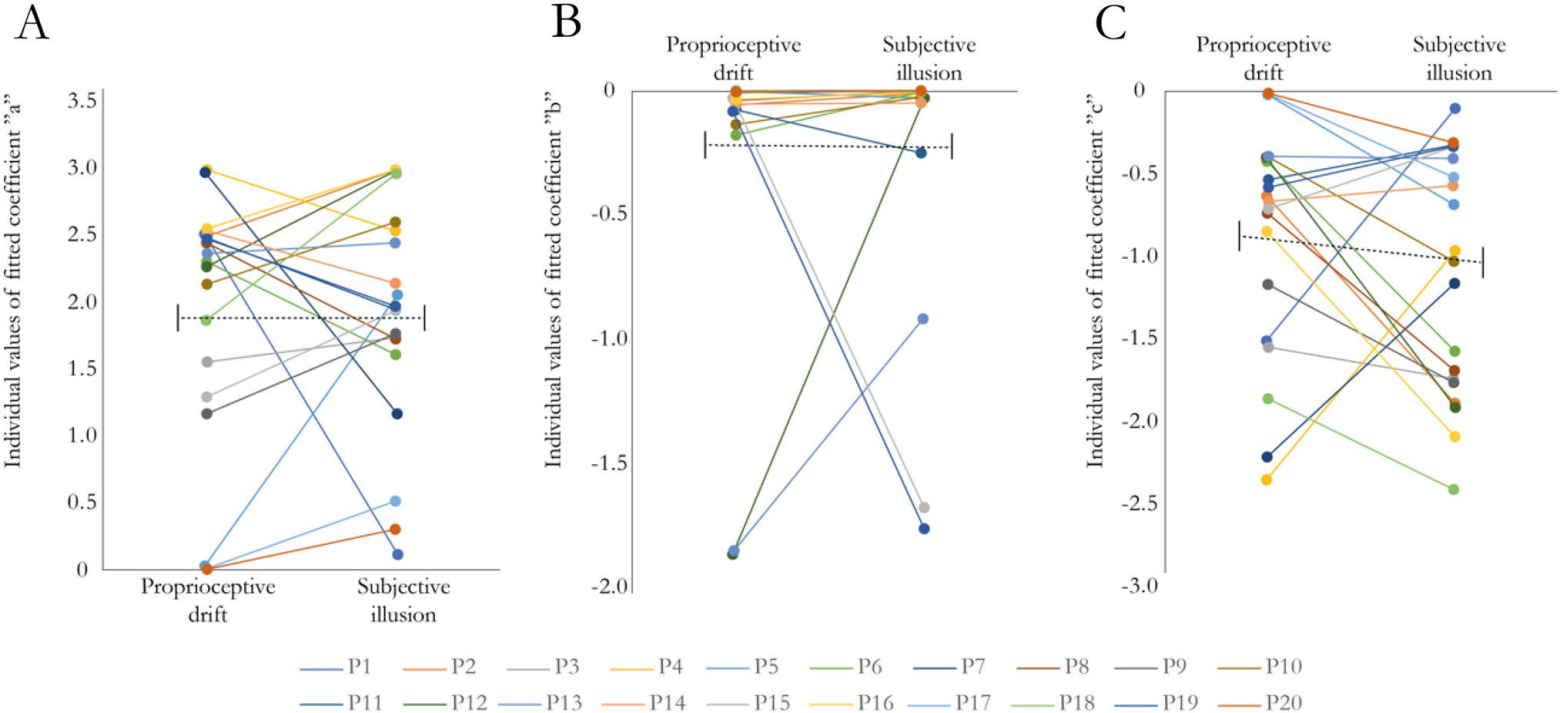
Comparison of the values of the coefficients for the fitted curves (a, b and c in the equation $y\, = \,a\, \times \,e^{bx} \, + \,c$) for the synchronous conditions in experiment 2. Panel A illustrates the coefficient “*a*” (intersect), panel B the coefficient “*b*” (decay) and panel C the coefficient “*c*” (constant). Individual data points and pairwise comparisons are shown for all participants (P1–P20). The black dotted lines illustrate mean and standard error of the mean (SEM). See Supplementary Fig. 4 for individual fitted curves and individual datapoints

To compare the decay curves of the Z-scored proprioceptive drift measure and the subjective illusion ratings, we analyzed the coefficients and the half-lives of the fitted exponential decay curves (Fig. 4, panel C and D and Fig. 6). The analysis of the curve fits (*R*^2^ values see supplementary Table 7) showed no significant main effect of outcome measure (*F* = 0.159, df = 17, *p* = 0.697, *η*^2^ = 0.003) or synchrony (*F* = 01.156, df = 17, *p* = 0.297, *η*^2^ = 0.023) and no significant interaction (*F* = 0.051, df = 17, *p* = 0.825, *η*^2^ < 0.0001). The analysis of the intersect values (‘a’ in the equation $y\, = \,a\, \times \,e^{bx} \, + \,c$) showed no significant main effect of outcome measure (*F* = 0.660, df = 17, *p* = 0.427, *η*^2^ = 0.027), no significant main effect of synchrony (*F* = 2.398, df = 17, *p* = 0.139, *η*^2^ = 0.027) or any significant interaction (*F* = 0.924, df = 17, *p* = 0.348, *η*^2^ = 0.015). The same pattern of results was seen for the decay coefficient (‘b’ in the equation $y\, = \,a\, \times \,e^{bx} \, + \,c$) with no main effect of outcome measure (*F* = 0.364, df = 17, *p* = 0.554, *η*^2^ = 0.005), synchrony (*F* = 0.463, df = 17, *p* = 0.505, *η*^2^ = 0.007) and no interaction (*F* = 0.362, df = 17, *p* = 0.555, *η*^2^ < 0.008), indicating that the decay of the fitted curves in both outcome measures had similar steepness and temporal dynamics. Finally, the constant values for the fitted exponential decay curves (‘c’ in the equation $y\, = \,a\, \times \,e^{bx} \, + \,c$) similarly showed no significant main effect of outcome measure (*F* = 0.095, df = 17, *p* = 0.762, *η*^2^ = 0.002), no significant main effect of synchrony (*F* = 2.139, df = 17, *p* = 0.160, *η*^2^ = 0.016) and no interaction (*F* = 1.129, df = 17, *p* = 0.301, *η*^2^ = 0.026). In an additional analysis, we calculated the half-lives of the fitted curves, which takes into account both the intersect, decay coefficient and constant of the exponential decay curve. The analysis revealed no significant main effect of outcome measure (*F* = 0.867, df = 17, *p* = 0.363, *η*^2^ = 0.014) but no main effect of synchrony (*F* = 1.102, df = 17, *p* = 0.307, *η*^2^ = 0.019) and no interaction (*F* = 0.912, df = 17, *p* = 0.352, *η*^2^ = 0.015). Bayesian hypothesis testing between the proprioceptive drift and subjective illusion ratings for each coefficient and half-life in the synchronous conditions showed weak to moderate support for the null hypothesis with Bayes factors (BF_10_) in the range 0.232–0.819 (Supplementary Table 3). The correlation analyses between the intersect, slope, constants and half-life of the curves for the proprioceptive drift and subjective illusion ratings showed no significant correlation (Supplementary Table 4).

In addition, we analyzed the synchronous–asynchronous differences for the two outcome measures. Curves were fitted to the z-score normalized data points as described in the methods section, and the coefficients of the resulting exponential curves on the form of $y\, = \,a\, \times \,e^{bx} \, + \,c$ were compared statistically. The analysis revealed no significant differences, with moderate support for the null hypothesis with Bayes factors (BF_10_) in the range 0.256–0.304 (Supplementary Fig. 2, Panel B, Supplementary Fig. 3, Panel B and Supplementary Table 5).

### Summary

The results from experiment 2 show a similar overall pattern as the findings from experiment 1. The rubber hand illusion displays substantial permanence after the brush stroking ceases, with both subjective ownership and proprioceptive drift thereafter displaying a relatively slow decay. The first step of the analysis indicates that the proprioceptive drift is maintained for 40 s after the end of the visuo-tactile stroking before tapering off. An unexpected difference in the proprioceptive drift at 300 s is probably related to a negative drift in the asynchronous condition at this timepoint. Since this finding was only observed at this timepoint and was not seen in experiment 1, and we have no explanation for it, we will not consider it further in this study. For the subjective ratings, we show that the significant difference in the feeling of ownership between the synchronous and asynchronous conditions persists for at least 60 s after the end of the visuo-tactile stimulation. However, we note that the absolute values of ownership ratings in the synchronous condition were lower in experiment 2 than in experiment 1. The ratings decreased to a mean value of 0 after 20 s, while in experiment 1, affirmative mean ratings (above 0) were observed even after 300 s (compare Fig. 2B and Fig. 5B). We speculate that this could be due to the reduced visual feedback from the rubber hand in experiment 2.

In the second step of the analysis, we demonstrate that the comparisons of the time courses and slopes of the curves fitted to the proprioceptive drift and subjective illusion ratings in experiment 2 show a significant main effect of outcome measure for the half time of the exponential decay curves, indicating that the decay of the subjective illusion ratings is slower than that for the proprioceptive drift. This difference, not observed in experiment 1, might relate to the differences in the experimental procedures discussed further below.

### Comparison between experiment 1 and experiment 2

To compare whether there were any differences between experiments 1 and 2 in terms of the effect of the amount of hand viewing time on the proprioceptive drift and subjective ratings, we first conducted a 2 × 2 × 6 ANOVA of the *z* score normalized values, with the factors synchrony (synchronous/asynchronous), experiment (experiment 1/experiment 2) and timing (0, 40, 60, 120, 300 s) for the proprioceptive drift measures and subjective illusion measures separately. For proprioceptive drift, we observed a main effect of experiment (*F* = 9.871, *p* = 0.002, *η*^2^ = 0.020) and a main effect of synchrony (*F* = 18.303, *p* < 0.001, *η*^2^ = 0.037) but no main effect of timing (*F* = 2.084, *p* = 0.066, *η*^2^ = 0.021) and no significant interactions. For the subjective illusion ratings, we similarly observed a main effect of experiment (*F* = 28.474, *p* < 0.001, *η*^2^ = 0.049) and a main effect of synchrony (*F* = 76.516, *p* < 0.001, *η*^2^ = 0.132) but no main effect of timing (*F* = 1.504, *p* = 0.187, *η*^2^ = 0.013) and significant interactions. The lack of significant interactions between the factors experiment and timing for both the proprioceptive drift (*F* = 0.159, *p* = 0.977, *η*^2^ = 0.002) and subjective illusion ratings (*F* = 0.349, *p* = 0.883, *η*^2^ = 0.003) indicates that the evolution of the measures is similar across experiments, whereas the lack of significant interactions between the factors experiment and synchrony for both the proprioceptive drift (*F* = 0.024, *p* = 0.877, *η*^2^ = 0.0005) and subjective illusion ratings (*F* = 0.727, *p* = 0.394, *η*^2^ = 0.001) indicates that the synchronous–asynchronous difference is consistent across experiments.

**Fig. 7:**
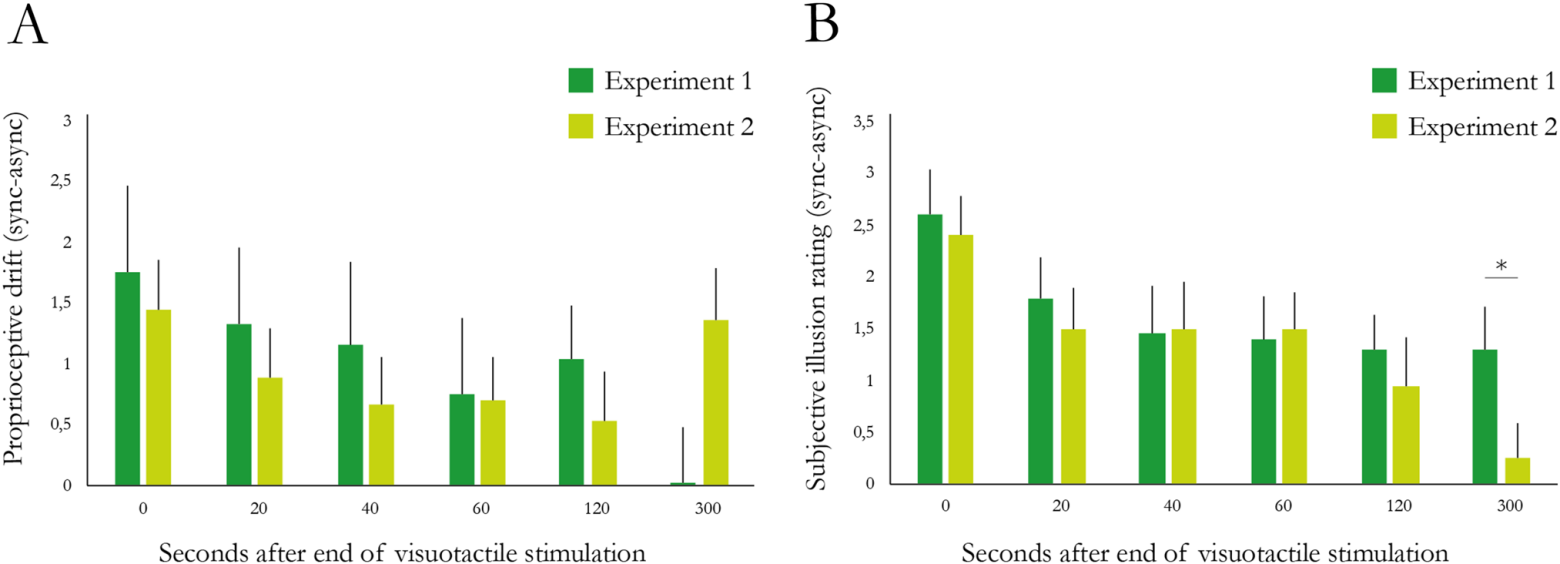
**A** Comparison between the differences in proprioceptive drift between synchronous and asynchronous conditions in experiment 1 and experiment 2. **B** Comparison between the differences in the subjective illusion ratings between synchronous and asynchronous conditions in experiment 1 and experiment 2. Error bars indicate SEMs. *indicates *p* < 0.05. *n* = 20 per experiment

To further analyze the differences, we conducted an independent sample interaction analysis, where the synchronous–asynchronous difference for the proprioceptive drift and subjective illusion ratings was calculated for each participant and each timepoint (0, 20, 40, 60, 120 and 300 s) in both experiments. We then compared each timepoint for both the proprioceptive drift and subjective rating in experiment 1 with its corresponding timepoint in experiment 2. Our results show that there is no difference in either the proprioceptive drift or the subjective ratings for timepoints 0, 20, 40, 60 and 120 s (Fig. 7). For the 300 s timepoint, the difference between synchronous and asynchronous subjective ratings is significantly larger in experiment 1 than in experiment 2 (*W* = 277.00, *p* = 0.034, rank-biserial correlation 0.385; Fig. 7, panel B), indicating that the illusion can be maintained longer if one can view the rubber hand in an anatomically congruent position for extended periods. The proprioceptive drift did not demonstrate any difference between the two experiments at this last timepoint (Supplementary Table 8).

## Discussion

We conducted two experiments to investigate the relationship between proprioceptive drift and the feeling of ownership in the rubber hand illusion. In particular, we investigated how these two measures of the rubber hand illusion decayed after the end of the visuo-tactile stimulation. There were two main findings. First, we found that the rubber hand illusion showed a substantial degree of persistence after the visuotactile stimulation stopped. In both experiments, we found that significant effects on subjective ownership ratings could be observed for up to 300 s and for proprioceptive drift up to 40 s after we stopped applying the brush strokes. Second, we found that the subjective feeling of ownership of the rubber hand, measured through a questionnaire statement, and the proprioceptive drift, registered with a hand localization task, had similar time courses for their reduction. Furthermore, regardless of whether the participants looked at the rubber hand for most of the time during the experiment (experiment 1) or kept their eyes closed as much as possible (experiment 2), we observed similar time courses in the reduction of subjective ownership and proprioceptive drift when comparing the synchronous condition to the asynchronous control. Collectively, these results suggest that once the perceptual systems have “decided” that the rubber hand is one’s own and that the visual impressions from the artificial hand and somatosensory sensations from the hidden real hand should be bound together, the brain tends to persist with this model in the absence of strong counterevidence against this interpretation. Only after tens of seconds is there evidence of a slow process of reverting this illusory perception back to veridical bodily awareness with the gradual loss of subjective ownership and decreasing proprioceptive drift.

Moreover, detailed analyses of the relative time courses of the reductions in proprioceptive drift and subjective ownership suggest that these processes depend at least in part on similar mechanisms, in contrast to our hypothesis (based on Abdulkarim and Ehrsson 2016). The curves for the decays of the proprioceptive drift and subjective illusion ratings are similar overall with no significant differences in the initial values (*z* score normalized values), slopes or half times. We do, however, observe a slower decay of the subjective illusion ratings in the second experiment, which seems to be driven by the asynchronous conditions and the inexplicable result at the 300 s timepoint. In a previous study (Abdulkarim and Ehrsson 2016), we showed that proprioceptive drift is not a causal prerequisite to the illusory experience in the rubber hand illusion, but we were open to the possibility of the causal relationship being directed in the opposite direction (i.e., the illusory experience leading to changes in hand position sense). The current findings, which show that the temporal dynamics for their reduction are similar, leave room for such an interpretation. The fact that subjective ownership and proprioceptive drift are related in the way the current data suggest is consistent with multisensory integration models of body ownership (see below).

According to the maximum likelihood estimation framework for multisensory integration, the integration of multiple senses into a coherent percept will be done by weighting the senses according to reliability (Ernst and Banks 2002). Of the senses involved in the rubber hand illusion and the proprioceptive drift (i.e., touch, vision and proprioception), vision typically has the highest reliability under good viewing conditions, and thus vision is often weighted highly in multisensory combination for bodily perception (Beers et al. 1999; Chancel et al. 2016; Ernst and Banks 2002; Reuschel et al. 2010; van Beers et al. 2002). With respect to our results, this could mean that the recalibration of hand position sense towards the rubber hand would occur as long as there is congruent visual input that supports the assumption that the participant’s hand is located at the position of the rubber hand, at least if vision is weighted very highly. However, our data show a slow reduction of the rubber hand illusion both in terms of proprioceptive drift and subjective ownership rather than stable maintenance. Moreover, the specific time courses of these reductions in drift and ownership were not significantly influenced by the amount of time participants were looking at the rubber hand (comparison of synchronous vs asynchronous conditions across experiments 1 and 2), which indicates that the loss of the illusion and reversal back to veridical bodily perception is driven by a process that does not depend on viewing time. However, we did observe significantly higher subjective illusion ratings and proprioceptive drift across all timepoints and conditions between the two experiments, which suggests that vision of the rubber hand did contribute to the multisensory processes but that the effect was relatively minor and not sufficient to maintain the rubber hand illusion in the absence of visuo-tactile correlations.

Existing theoretical models of body ownership have not focused on what would happen during the spontaneous decay of the rubber hand illusion but have rather been centered on the factors responsible for the elicitation of the rubber hand illusion. In the simple connectionist model proposed by Botvinick and Cohen, the illusion is thought of as a three-way interaction between vision, touch and proprioception (Botvinick and Cohen, 1998). This model does not make any predictions with respect to time courses, nature of the interactions, or the relative weighting of the different senses, but our data are broadly consistent with this model, as both subjective ownership and drift showed changes in the same direction. Tsakiris et al. proposed a model in which the rubber hand illusion emerges following three so-called critical comparisons between the visual form, posture and sensory inputs from the rubber hand and the participants’ real hand (Tsakiris 2010). The model then emphasizes that the resulting referral of tactile sensation to the rubber hand rises to the subjective experience of body ownership. This model does not make any explicit predictions about what would happen when visuo-tactile stimulation stops, so the implications of our data for this model are unclear. The model by Makin et al. emphasizes that visuotactile integration within peripersonal space drives the illusion, which further gives rise to the recalibration of hand position sense towards the rubber hand illusion (Makin et al. 2008). This model supports the notion that proprioceptive drift is not causally necessary for the rubber hand illusion but rather is a consequence of the illusion itself. The model focuses on the importance of referral of touch and visuo-tactile integration in peri-personal space and does not explicitly specify what would happen when the visuo-tactile stimulation stops, although a slow shift in peri-personal space back towards the location of the real hand is consistent with this model.

Recent multisensory integration models (Ehrsson and Chancel 2019; Ehrsson 2020; Fang et al. 2019; Kilteni et al. 2015; Körding et al. 2007; Samad et al. 2015) emphasize the rubber hand illusion as a multisensory binding problem, which the brain solves by a process of “causal inference” in which the probability that sensory signals share a common cause is computed (Körding et al. 2007). According to this theory, the most likely causal structure of the visual and somatosensory signals is inferred based on spatial proximity, simultaneity, temporal correlation, and prior experiences. Based on probabilistic computations, the multisensory signals are either combined (rubber hand illusion) or segregated (rubber hand and real hand perceived as different objects). In this framework, subjective ownership is intimately related to causal inference (multisensory binding) and the proprioceptive drift to the combination of vision and proprioception. The model is flexible and can incorporate many different kinds of sensory input (Ehrsson and Chancel 2019) and does not emphasize the referral of touch as much as some of the earlier models (Makin et al. 2008; Tsakiris 2010). However, the causal inference model has only been empirically validated in cases of multisensory integration, whereas our current study is more related to the gradual loss of integration, in which already inferred (illusory) common cause is slowly “reevaluated” into distinct causes. According to this model, once the rubber hand illusion has been elicited, the rubber hand is represented as one’s own hand, i.e., the owned rubber hand is the single cause for both vision and somatosensation of one’s hand. This can explain the prolonged illusory experience after the end of the visuo-tactile stimulation we observe in our data. In the absence of strong evidence against the interpretation that the rubber hand is one’s own, the illusion is maintained.

The question then arises of why we observe a degradation of the illusion over time at all. One possibility is that the prior probability of the rubber hand being one’s own is lower than the “default prior” of the rubber hand being an external object, so without clear sensory evidence in support of the former, the multisensory causal inference process will gradually reverse back to the more likely default experience, and the ownership of the rubber hand is lost. Alternatively, continuous afferent signals from muscles and joints in the real hand and arm provide subtle evidence against the ownership of the rubber hand, because even though the rubber hand is placed in close proximity to the real hand (15 cm), within peripersonal space (Brozzoli et al. 2012; Lloyd, 2007), and in a matching orientation (Ehrsson et al. 2004; Ide 2013), there is still a spatial disparity between proprioception and vision, and even small disparities between vision and proprioception influence the rubber hand illusion (Chancel and Ehrsson 2020). Given that the proprioceptive afferent signals from the relaxed real arm are relatively weak (at least compared to the feedback during movement) and that the visuo-proprioceptive mismatch is relatively subtle, it might take some time to accumulate enough sensory evidence against the interpretation that the rubber hand is one’s own. Our observation of the similarity in the time course of the gradual loss of illusory subjective rubber hand ownership and proprioceptive drift is consistent with this probabilistic multisensory integration framework. The weaker the subjective illusion and the causal inference that the rubber hand is one’s own gets over time, the less the visual and proprioceptive signals should be combined, and hence the weaker the proprioceptive drift; this is what we observe in our data.

From perception research, we know that once an illusion or stimulus stops after a period of adaptation, there tends to be an aftereffect directed in the opposite direction to the illusion or stimulus presented. This has been shown, for example, in unimodal visual (Bednar 2013; Fernández-Ruiz et al. 2004) and proprioceptive illusions (Goodwin et al. 1972; Kito et al. 2006; Perasso et al. 2019; Seizova-Cajic et al. 2007), as well as in multisensory illusions, such as the ventriloquism aftereffect (Sato et al. 2007) or the face recognition aftereffect (Pond et al. 2013). This study does not examine the aftereffect of the rubber hand illusion per se; rather, it examines the perseverance of two commonly used illusion measures after the end of the visuotactile stimulation. Hence, we refrain from calling our current key results rubber hand illusion “aftereffects”, since what we examine in this study is the permanence of a multisensory illusion following the end of the synchronous visuo-tactile stimulation and not perceptual effects in the opposite direction after removal of the perceptual manipulation in question.

In conclusion, the present results highlight previously unknown temporal properties of the rubber hand illusion and reveal new insights into the relationship between proprioceptive drift and subjective illusion ratings. Knowledge about these temporal relationships provides valuable new information for neurocognitive models of body ownership. Furthermore, knowledge about the persistence and slow decay of the rubber hand illusion after visuotactile stimulation stops is relevant for the design of efficient and well-planned studies, for example, emphasizing the need to consider possible carryover effects that might arise from a sustained feeling of ownership across trials. Moreover, the sustained nature of the rubber hand illusion is also good news for experimenters who want to present various tests after the visuotactile stimulation stops, for example, reaction time tasks (Reader et al. 2020) or registration of threat-evoked SCR responses (Guterstam et al. 2019; Petkova and Ehrsson 2009), as our data suggest that the time windows of one to ten seconds that are often used are well within a period when the illusion is still maintained. Finally, our results have a bearing on applied neuroscience and the development of advanced prosthetic limbs that feel more like real limbs (Collins et al. 2017; Ehrsson et al. 2008). The sustained nature of the ownership illusion means that it will not be necessary to provide constant visuotactile stimulation in such prosthetic devices to maintain a sense of embodiment, a constraint that otherwise has been considered a notable limitation (Zbinden et al. 2021). We speculate that the illusion of the prosthetic limb as part of one’s own body could be maintained for at least 40 s, after which a few “booster” congruent multisensory stimulations may be sufficient to re-elicit the illusion to full strength; however, this will need to be investigated in future studies.

## Supplementary Information

Below is the link to the electronic supplementary material.

Supplementary file1 (XLSX 17 KB)

Supplementary file2 (DOCX 8645 KB)

## Data Availability

The source data analyzed during the current study are made publicly available as supplementary material.
