# Supplementary material for: Sustained rubber hand illusion after the end of visuotactile stimulation with a similar time course for the reduction of subjective ownership and proprioceptive drift
Source: Exp Brain Res. 2021 Sep 15;239(12):3471–86. doi: 10.1007/s00221-021-06211-8 (PMC8599369; doi:10.1007/s00221-021-06211-8)
Supplement: Supplementary file 2 — Supplementary file2 (DOCX 8645 KB) [file 221_2021_6211_MOESM2_ESM.docx]

# Supplementary material

**Supplementary table 1**. The statistics for the comparisons between the synchronous and asynchronous conditions for both the subjective illusion ratings and the proprioceptive drift in experiment 1. SIRs= Subjective Illusion Ratings in synchronous condition, SIRas= Subjective Illusion Ratings in asynchronous condition, PDs= Proprioceptive Drift in synchronous condition, PDas= Proprioceptive Drift in asynchronous condition.

| **Proprioceptive drift** | | | | | | | | | | | | | |
| --- | --- | --- | --- | --- | --- | --- | --- | --- | --- | --- | --- | --- | --- |
|  | |  | |  | | **t** | | **df** | | **p** | | **Cohen's d** | |
| PDs 0 |  | - |  | PDas 0 |  | 2.442 |  | 19 |  | 0.025 |  | 0.546 |  |
| PDs 20 |  | - |  | PDas 20 |  | 2.157 |  | 19 |  | 0.044 |  | 0.482 |  |
| PDs 40 |  | - |  | PDas 40 |  | 1.717 |  | 19 |  | 0.102 |  | 0.384 |  |
| PDs 60 |  | - |  | PDas 60 |  | 1.182 |  | 19 |  | 0.252 |  | 0.264 |  |
| PDs 120 |  | - |  | PDas 120 |  | 2.334 |  | 19 |  | 0.031 |  | 0.522 |  |
| PDs 300 |  | - |  | PDas 300 |  | 0.058 |  | 19 |  | 0.955 |  | 0.013 |  |
|  | | | | | | | | | | | | | |
| *Note.*  Student's t-test. | | | | | | | | | | | | | |

| **Subjective illusion ratings** | | | | | | | | | | | | | |
| --- | --- | --- | --- | --- | --- | --- | --- | --- | --- | --- | --- | --- | --- |
|  | |  | |  | | **t** | | **df** | | **p** | | **Cohen's d** | |
| SIRs 0 |  | - |  | SIRas 0 |  | 6.110 |  | 19 |  | < .001 |  | 1.366 |  |
| SIRs 20 |  | - |  | SIRas 20 |  | 4.485 |  | 19 |  | < .001 |  | 1.003 |  |
| SIRs 40 |  | - |  | SIRas 40 |  | 3.265 |  | 19 |  | 0.004 |  | 0.730 |  |
| SIRs 60 |  | - |  | SIRas 60 |  | 3.290 |  | 19 |  | 0.004 |  | 0.736 |  |
| SIRs 120 |  | - |  | SIRas 120 |  | 3.728 |  | 19 |  | 0.001 |  | 0.834 |  |
| SIRs 300 |  | - |  | SIRas 300 |  | 3.115 |  | 19 |  | 0.006 |  | 0.696 |  |
|  | | | | | | | | | | | | | |
| *Note.*  Student's t-test. | | | | | | | | | | | | | |

**Supplementary table 2**. R^2^ values and standard deviation for the fitted curves for the outcome measures and conditions in experiment 1. PD= proprioceptive drift. SIR= subjective illusion ratings.

| **Fitted curve** | **Mean R^2^** | **Standard deviation** |
| --- | --- | --- |
| PD synchronous | 0,54 | 0,31 |
| PD asynchronous | 0,40 | 0,32 |
| SIR synchronous | 0,56 | 0,37 |
| SIR asynchronous | 0,57 | 0,36 |

**Supplementary table 3.** Bayesian hypothesis testing between the proprioceptive drift and subjective illusion ratings for each coefficient in the fitted curves in the synchronous condition. PD = proprioceptive drift, SIR=subjective illusion ratings. “a”, “b” and “c” denote the coefficient in the fitted curve *y=a * e^bx^ + c,* whereas “50%” indicates the half-life of the z score normalized values.

**Experiment 1**

| **Bayesian Paired Samples T-Test** | | | | | | | | | |
| --- | --- | --- | --- | --- | --- | --- | --- | --- | --- |
| Measure 1 | |  | | Measure 2 | | BF₁₀ | | error % | |
| PD a |  | - |  | SIR a |  | 0.328 |  | 0.019 |  |
| PD b |  | - |  | SIR b |  | 0.233 |  | 0.022 |  |
| PD c |  | - |  | SIR c |  | 0.382 |  | 0.016 |  |
| PD 50% |  | - |  | SIR 50% |  | 0.337 |  | 0.019 |  |
|  | | | | | | | | | |

**Experiment 2**

| **Bayesian Paired Samples T-Test** | | | | | | | | | |
| --- | --- | --- | --- | --- | --- | --- | --- | --- | --- |
| Measure 1 | |  | | Measure 2 | | BF₁₀ | | error % | |
| PD a |  | - |  | SIR a |  | 0.232 |  | 0.022 |  |
| PD b |  | - |  | SIR b |  | 0.234 |  | 0.022 |  |
| PD c |  | - |  | SIR c |  | 0.469 |  | 0.012 |  |
| PD 50% |  | - |  | SIR 50% |  | 0.819 |  | 0.002 |  |
|  | | | | | | | | | |

**Supplementary table 4.** Correlation between each coefficient in the fitted curve *y=a * e^bx^ + c* as well as the half-life of the proprioceptive drift and subjective illusion ratings in the synchronous condition.

**Experiment 1**

| **coefficient** | **r-value (Pearson correlation)** | **p-value** |
| --- | --- | --- |
| a | -0.141 | 0.552 |
| b | -0.066 | 0.781 |
| c | -0.045 | 0.852 |
| half-time | -0.130 | 0.585 |

**Experiment 2**

| **coefficient** | **r-value (Pearson correlation)** | **p-value** |
| --- | --- | --- |
| a | 0.379 | 0.099 |
| b | 0.132 | 0.580 |
| c | 0.261 | 0.267 |
| half-time | -0.008 | 0.973 |


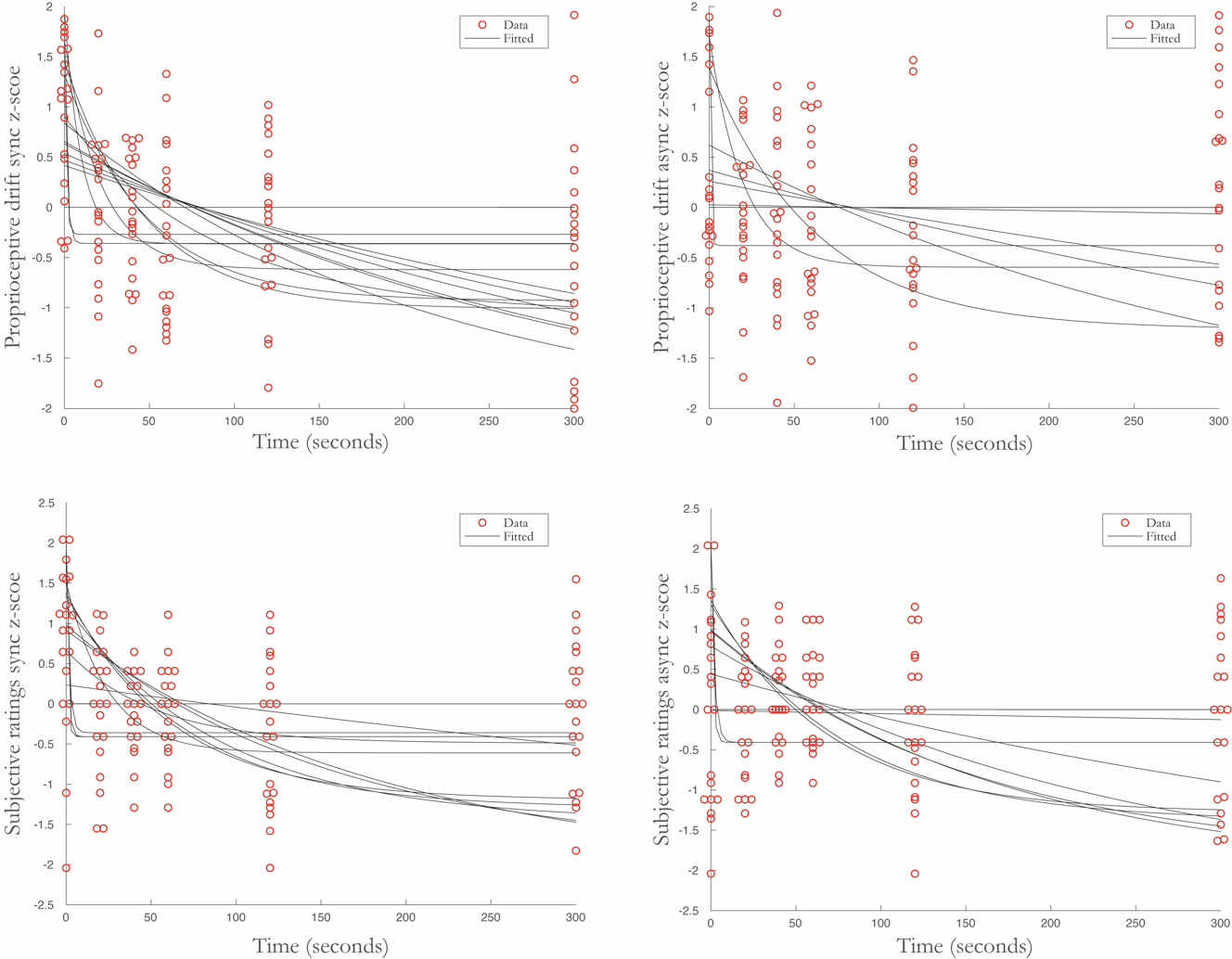


**Supplementary Figure 1.** The individual data points and fitted curves for each participant in experiment 1. The four panels show each condition and outcome measure. Sync=synchronous, async=asynchronous, n=20. Note that some of the fitted curves are overlapping and hence not visible in the figures.


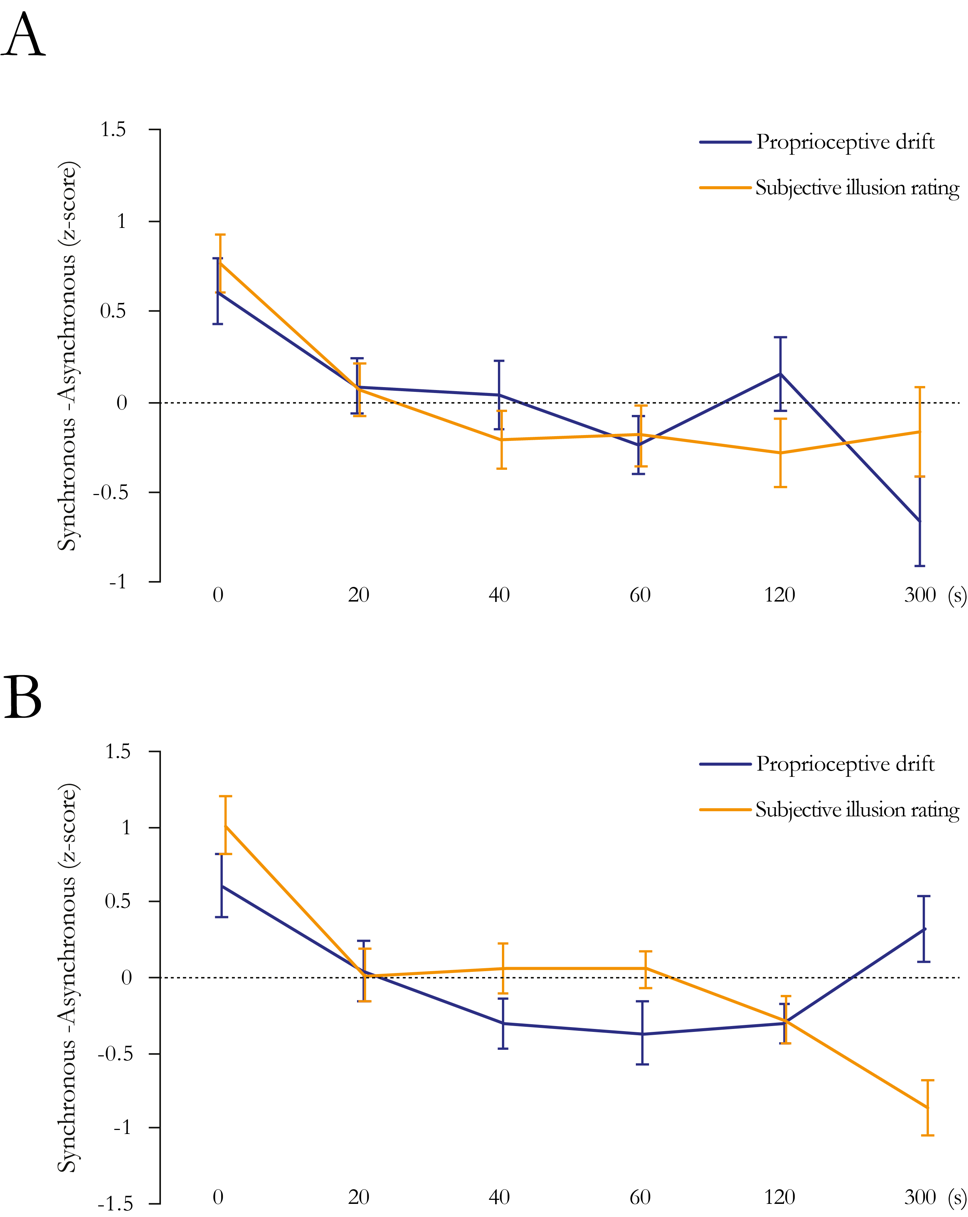


**Supplementary figure 2.** The z-score normalized synchronous-asynchronous difference for the proprioceptive drift and subjective illusion ratings in experiment 1 (A) and experiment 2 (B). Error bars indicate standard error of the mean (SEM), n=20.


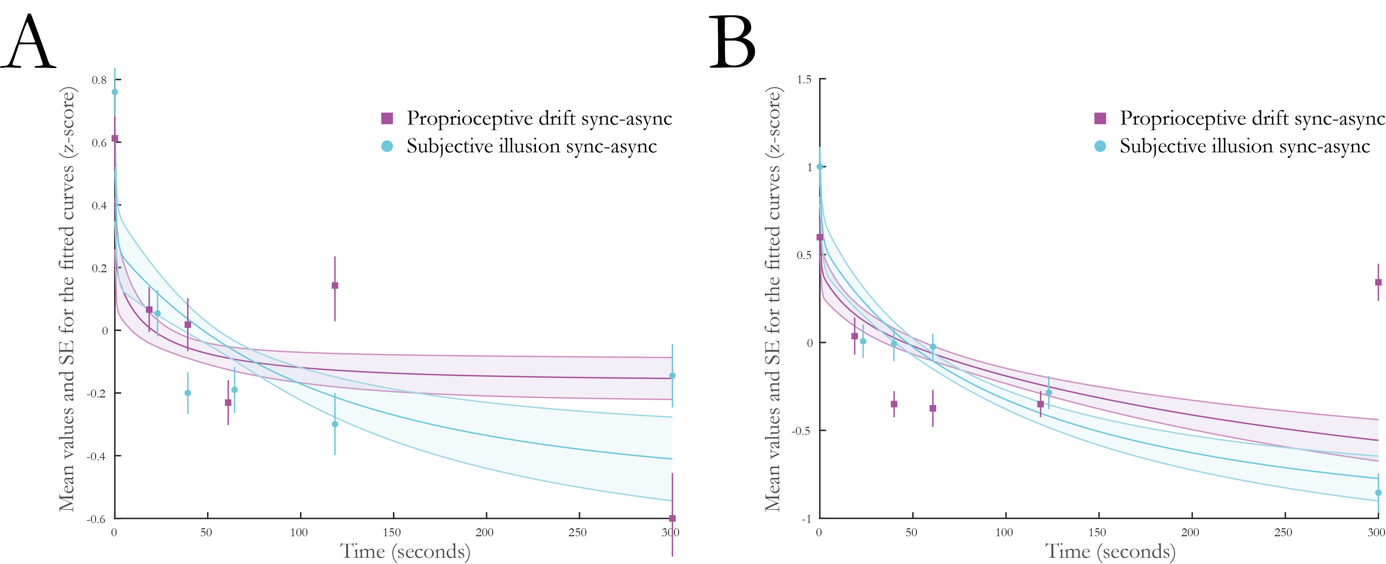


**Supplementary Figure 3.** Mean fitted curves for synchronous-asynchronous difference in experiment 1 (A) and 2 (B). The exponential curves represent the mean fitted curve (averaged fitted values) and the shaded areas around them indicate the standard errors. The squares and dots indicate the mean z-scores for each of the six measured timepoints (0, 20, 40, 60, 120 and 300 seconds), with the error bars indicating the standard error of the mean, n=20.

**Supplementary table 5.** Comparison between the coefficients of the fitted curves (*y=a * e^bx^ + c)* for the z-score normalized synchronous-asynchronous difference for the proprioceptive drift and subjective illusion ratings. PD = proprioceptive drift, SIR=subjective illusion ratings.

**Experiment 1**

| **Paired Samples T-Test (Student’s)** | | | | | | | | | | | | | |
| --- | --- | --- | --- | --- | --- | --- | --- | --- | --- | --- | --- | --- | --- |
| Measure 1 | |  | | Measure 2 | | t | | df | | p | | Cohen's d | |
| PD a |  | - |  | SIR a |  | -0.584 |  | 19 |  | 0.566 |  | -0.131 |  |
| PD b |  | - |  | SIR b |  | -0.999 |  | 19 |  | 0.330 |  | -0.223 |  |
| PD c |  | - |  | SIR c |  | 0.407 |  | 19 |  | 0.688 |  | 0.091 |  |
|  | | | | | | | | | | | | | |

| **Bayesian Paired Samples T-Test** | | | | | | | | | |
| --- | --- | --- | --- | --- | --- | --- | --- | --- | --- |
| Measure 1 | |  | | Measure 2 | | BF₁₀ | | error % | |
| PD_a |  | - |  | LS_a |  | 0.271 |  | 0.021 |  |
| PD_b |  | - |  | LS_b |  | 0.361 |  | 0.018 |  |
| PD_c |  | - |  | LS_c |  | 0.250 |  | 0.022 |  |
|  | | | | | | | | | |

**Experiment 2**

| **Paired Samples T-Test (Student’s)** | | | | | | | | | | | | | |
| --- | --- | --- | --- | --- | --- | --- | --- | --- | --- | --- | --- | --- | --- |
| Measure 1 | |  | | Measure 2 | | t | | df | | p | | Cohen's d | |
| PD a |  | - |  | SIR a |  | -0.463 |  | 19 |  | 0.648 |  | -0.104 |  |
| PD b |  | - |  | SIR b |  | -0.777 |  | 19 |  | 0.447 |  | -0.174 |  |
| PD c |  | - |  | SIR c |  | -0.559 |  | 19 |  | 0.582 |  | -0.125 |  |
|  | | | | | | | | | | | | | |

| **Bayesian Paired Samples T-Test** | | | | | | | | | |
| --- | --- | --- | --- | --- | --- | --- | --- | --- | --- |
| Measure 1 | |  | | Measure 2 | | BF₁₀ | | error % | |
| PD a |  | - |  | SIR a |  | 0.256 |  | 0.021 |  |
| PD b |  | - |  | SIR b |  | 0.304 |  | 0.020 |  |
| PD c |  | - |  | SIR c |  | 0.267 |  | 0.021 |  |
|  | | | | | | | | | |

**Supplementary table 6**. The statistics for the comparisons between the synchronous and asynchronous conditions for both the subjective illusion ratings and the proprioceptive drift in experiment 2. SIRs= Subjective Illusion Ratings in synchronous condition, SIRas= Subjective Illusion Ratings in asynchronous condition, PDs= Proprioceptive Drift in synchronous condition, PDas= Proprioceptive Drift in asynchronous condition.

| **Proprioceptive drift** | | | | | | | | | | | | | |
| --- | --- | --- | --- | --- | --- | --- | --- | --- | --- | --- | --- | --- | --- |
|  | |  | |  | | **t** | | **df** | | **p** | | **Cohen's d** | |
| PDs 0 |  | - |  | PDas 0 |  | 3.463 |  | 19 |  | 0.003 |  | 0.774 |  |
| PDs 20 |  | - |  | PDas 20 |  | 2.179 |  | 19 |  | 0.042 |  | 0.487 |  |
| PDs 40 |  | - |  | PDas 40 |  | 1.721 |  | 19 |  | 0.101 |  | 0.385 |  |
| PDs 60 |  | - |  | PDas 60 |  | 1.933 |  | 19 |  | 0.068 |  | 0.432 |  |
| PDs 120 |  | - |  | PDas 120 |  | 1.298 |  | 19 |  | 0.210 |  | 0.290 |  |
| PDs 300 |  | - |  | PDas 300 |  | 3.239 |  | 19 |  | 0.004 |  | 0.724 |  |
|  | | | | | | | | | | | | | |
| *Note.*  Student's t-test. | | | | | | | | | | | | | |

| **Subjective illusion ratings** | | | | | | | | | | | | | |
| --- | --- | --- | --- | --- | --- | --- | --- | --- | --- | --- | --- | --- | --- |
|  | |  | |  | | **t** | | **df** | | **p** | | **Cohen's d** | |
| SIRs 0 |  | - |  | SIRas 0 |  | 6.439 |  | 19 |  | < .001 |  | 1.440 |  |
| SIRs 20 |  | - |  | SIRas 20 |  | 3.807 |  | 19 |  | 0.001 |  | 0.851 |  |
| SIRs 40 |  | - |  | SIRas 40 |  | 3.249 |  | 19 |  | 0.004 |  | 0.726 |  |
| SIRs 60 |  | - |  | SIRas 60 |  | 4.094 |  | 19 |  | < .001 |  | 0.916 |  |
| SIRs 120 |  | - |  | SIRas 120 |  | 2.009 |  | 19 |  | 0.059 |  | 0.449 |  |
| SIRs 300 |  | - |  | SIRas 300 |  | 0.773 |  | 19 |  | 0.449 |  | 0.173 |  |
|  | | | | | | | | | | | | | |
| *Note.*  Student's t-test. | | | | | | | | | | | | | |

**Supplementary Table 7.** R^2^ values and standard deviation for the fitted curves for the outcome measures and conditions in experiment 2. PD= proprioceptive drift. SIR= subjective illusion ratings.

| **Fitted curve** | **Mean R^2^** | **Standard deviation** |
| --- | --- | --- |
| PD synchronous | 0,63 | 0,35 |
| PD asynchronous | 0,52 | 0,25 |
| SIR synchronous | 0,63 | 0,31 |
| SIR asynchronous | 0,57 | 0,34 |


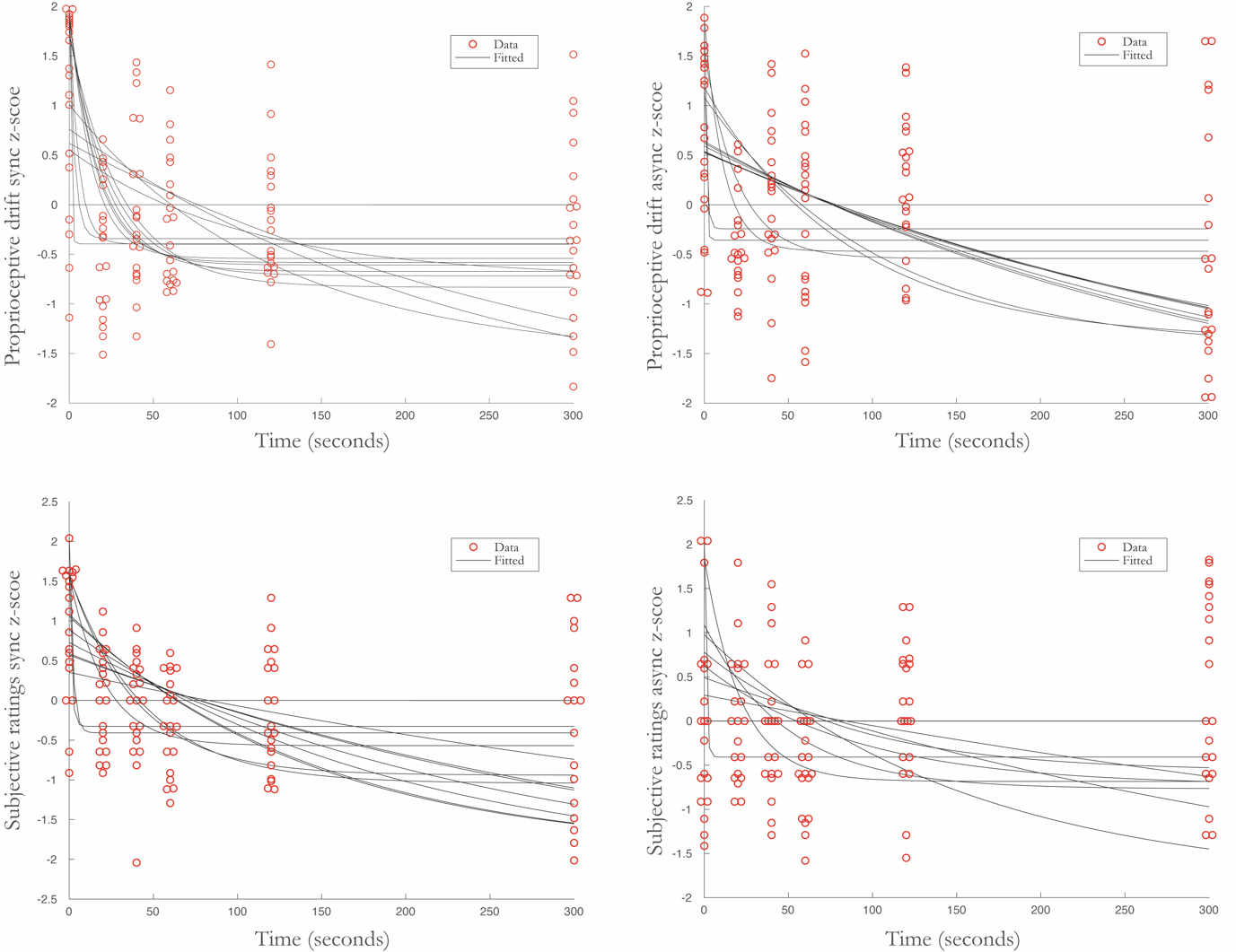


**Supplementary Figure 4.** The individual data points and fitted curves for each participant in experiment 2. The four panels show each condition and outcome measure. Sync=synchronous, async=asynchronous, n=20. Note that some of the fitted curves are overlapping and hence not visible in the figures.

**Supplementary table 8**. The statistics for the comparisons of the synchronous-asynchronous difference for the proprioceptive drift and subjective illusion ratings between experiments 1 & 2. E1= experiment 1, E2= experiment 2, SIR= Subjective Illusion Ratings, PD= Proprioceptive Drift.

| **Independent Samples T-Test** | | | | | | | | | |
| --- | --- | --- | --- | --- | --- | --- | --- | --- | --- |
|  | | **W** | |  | | **p** | | **Rank-Biserial Correlation** | |
| E1/E2 PD 0 |  | 198.500 |  |  |  | 0.978 |  | -0.007 |  |
| E1/E2 PD 20 |  | 206.000 |  |  |  | 0.882 |  | 0.030 |  |
| E1/E2 PD 40 |  | 202.000 |  |  |  | 0.968 |  | 0.010 |  |
| E1/E2 PD 60 |  | 176.500 |  |  |  | 0.534 |  | -0.117 |  |
| E1/E2 PD 120 |  | 211.500 |  |  |  | 0.766 |  | 0.058 |  |
| E1/E2 PD 300 |  | 133.000 |  |  |  | 0.072 |  | -0.335 |  |
| E1/E2 SIR 0 |  | 208.000 |  |  |  | 0.837 |  | 0.040 |  |
| E1/E2 SIR 20 |  | 215.000 |  |  |  | 0.690 |  | 0.075 |  |
| E1/E2 SIR 40 |  | 197.500 |  |  |  | 0.956 |  | -0.012 |  |
| E1/E2 SIR 60 |  | 192.000 |  |  |  | 0.836 |  | -0.040 |  |
| E1/E2 SIR 120 |  | 226.500 |  |  |  | 0.467 |  | 0.133 |  |
| E1/E2 SIR 300 |  | 277.000 |  |  |  | 0.034 |  | 0.385 |  |
|  | | | | | | | | | |
| *Note.*  Mann-Whitney U test. | | | | | | | | | |
